# Identification and Expression Analysis of G-Protein-Coupled Receptors Provide Insights into Functional and Mechanistic Responses to Herbivore-Induced Plant Volatiles of *Paracarophenax alternatus*

**DOI:** 10.3390/ijms26125890

**Published:** 2025-06-19

**Authors:** Ruiheng Lin, Xu Chu, Yangming Zhang, Sikai Ke, Yunfeng Zheng, Wei Yu, Feiping Zhang, Songqing Wu

**Affiliations:** 1 State Key Laboratory of Agricultural and Forestry Biosecurity, College of Forestry, Fujian Agriculture and Forestry University, Fuzhou 350002, China; 2 Key Laboratory of Integrated Pest Management in Ecological Forests, Fujian Agriculture and Forestry University, Fuzhou 350002, China; 3 Jinshan College of Fujian Agriculture, Forestry University, Fuzhou 350002, China; 4 Guizhou Institute of Biology, Guizhou Academy of Sciences, Guiyang 550009, China; 5 Conservation and Utilization of Natural Biological Resources, Fujian Provincial University Engineering Center, Fujian Agriculture and Forestry University, Fuzhou 350002, China

**Keywords:** *Paracarophenax alternatus*, biocontrol, *Monochamus alternatus*, G-protein-coupled receptor, pine wilt disease

## Abstract

Herbivore-induced plant volatiles (HIPVs) play a pivotal role in mediating tritrophic interactions between plants, herbivores, and their natural enemies. *Paracarophenax alternatus*, a parasitic mite targeting the egg stage of *Monochamus alternatus*, has emerged as a promising biocontrol agent. However, its ability to detect *Pinus massoniana*-derived HIPVs for host insect localization remains unclear. G-protein-coupled receptors (GPCRs) may play a role in mediating the perception of HIPVs and associated chemosensory signaling pathways in mites. In this study, a total of 85 GPCRs were identified from *P. alternatus.* All GPCRs exhibited conserved transmembrane domains and stage-specific expression patterns, with 21 receptors significantly upregulated in viviparous mites. Combined with two previously identified odorant receptors (ORs), six candidate chemosensory receptors were selected for molecular dynamics simulations to validate their binding stability with key volatile compounds. The results demonstrate that specific GPCRs likely facilitate HIPV detection in mites, enabling precise host localization within dynamic ecological niches. Our findings provide critical insights into the molecular basis of mite–host interactions and establish a framework for optimizing *P. alternatus*-based biocontrol strategies against pine wilt disease vectors.

## 1. Introduction

Pine forests significantly contribute to carbon storage in the Northern Hemisphere. However, the rising mortality rates of pine trees caused by pathogens and pests pose a substantial threat to global carbon sinks and biomass [1,2]. Among them, *Monochamus alternatus* (Hope, 1842), serving as the primary vector insect for *Bursaphelenchus xylophilus* ((Steiner & Buhrer, 1934) Nickle, 1970), exhibits substantial dispersal risks, and the challenges in its control and prevention remain critically severe [3,4]. To facilitate sustainable forest management, there is a pressing need to develop advanced, safe, and durable biological control strategies. Recently, the application of predatory mites to sustainably suppress pest population densities has emerged as a pivotal green control approach, offering substantial ecological and economic benefits [5,6]. *Paracarophenax alternatus* (Xu & Zhang, 2018) is currently the only reported parasitic natural enemy mite targeting the egg stage of *M. alternatus*. It is considered an exceptionally promising biocontrol agent due to its strong parasitization ability and short reproductive cycle [7,8]. Notably, the unique dispersal mechanism of *P. alternatus* promotes cross-species dispersal among beetles, which is critical for ensuring population persistence and enhancing biocontrol efficacy [9,10].

Chemical communication plays a pivotal role in mediating tritrophic interactions among plants, herbivores, and predatory mites. Natural enemy mites can precisely locate hosts by detecting herbivore-induced plant volatiles (HIPVs) [11]. Mite detection of HIPVs involves a sophisticated olfactory mechanism requiring coordinated participation of multiple proteins [12,13]. Beyond olfactory-related proteins traditionally considered central to olfaction, numerous G-protein-coupled receptors (GPCRs) have been implicated in chemosensory processes [14,15]. Despite existing studies proposing that components of the GPCR pathway may participate in predatory mite olfaction [16,17], the mechanistic basis of odorant signal transduction in predatory mites remains fundamentally unresolved. GPCRs are highly conserved proteins characterized by a transmembrane structure composed of seven α-helical segments. Based on sequence, structure, and function, they are classified into four families: rhodopsin-like receptors (Family A), secretin-like receptors (Family B), metabotropic glutamate receptors (Family C), and frizzled/smoothened receptors (Family F) [18,19]. GPCRs transduce extracellular signals—such as hormones, neurotransmitters, and environmental stimuli—into secondary intracellular second messengers via coupling with heterotrimeric G-proteins and downstream effectors, thereby regulating essential physiological responses in insects [20]. Recent advances in bioinformatics and high-throughput sequencing have accelerated GPCR identification and functional annotation. A total of 65 neuropeptide GPCR genes were identified in spider mites [21], while 200, 276, and 102 GPCR genes were characterized in *Drosophila melanogaster* [22,23], *Anopheles gambiae* [24], and *Rhopalosiphum padi* [25], respectively. Moreover, the functions of GPCRs have been explored extensively in several insects. For example, multiple GPCRs have been shown to regulate vitellogenesis and oocyte maturation in *Locusta migratoria* [26]. Dopamine receptors regulate aversive olfactory memory formation in *D. melanogaster* [27]. In *Hyphantria cunea*, the tachykinin receptor affects starvation tolerance and feeding behavior [28]. However, the involvement of GPCRs in *P. alternatus* has not yet been elucidated.

Reverse transcription–quantitative polymerase chain reaction (RT-qPCR) is a highly sensitive and rapid analytical technique extensively employed in molecular biology research to achieve precise quantification of target genes’ expression levels across diverse biological conditions [29]. The continuous advancement of protein prediction technologies has accelerated the advancement of structural bioinformatics [30]. Molecular docking has been extensively applied in the study of insect chemosensory systems to identify ligand molecules capable of binding with target proteins, thereby elucidating the mechanisms of molecular interactions and signal transduction pathways [31,32]. In this study, we identified the GPCR gene family in *P. alternatus* through transcriptome sequencing. The phylogenetic relationship and structure domains of the putative candidate GPCRs were analyzed by constructing a phylogenetic tree and performing bioinformatics analysis. Moreover, RT-qPCR was employed to determine the expression levels of GPCR genes under varying physiological states. The results are expected to provide relevant information for further functional studies in *P. alternatus*. Subsequently, integrated homology modeling and molecular docking were conducted to investigate interactions between GPCR receptors and key HIPV ligands emitted by *Pinus massoniana* Lamb.

This study aims to provide important insights for elucidating the biological functions of GPCRs in *P. alternatus* while establishing a theoretical foundation for developing novel biocontrol strategies targeting the egg stage of *M. alternatus*, thus contributing to the prevention of pine wilt disease. Moreover, it provides a theoretical foundation for unraveling tritrophic interactions among *P. massoniana*, *M. alternatus*, and *P. alternatus*.

## 2. Results

### 2.1. Identification and Phylogenetic Analysis of GPCR Genes in P. alternatus

GPCR genes from the seven mites described above were used as reference sequences. A total of 85 GPCRs were identified from *P. alternatus* through a search of our own transcriptome data. Among these GPCRs, 64 receptors belonged to Family A, 12 receptors belonged to Family B, 5 receptors belonged to Family C, and 4 receptors belonged to Family F. Family A of *P. alternatus* GPCRs consisted of 4 opsin, 26 biogenic amine receptors, and 34 neuropeptide and protein hormone receptors. The lengths of amino acid sequences encoded by the identified GPCR genes ranged from 88 to 1949 residues (Table 1).

Among Family A members, we identified two rhodopsins (PaltRh2-1 and PaltRh2-2), which clustered phylogenetically with the *D. tinctorium* rhodopsin (RWS03304.1). Additionally, two odorant receptors (ORs) were identified. Although ORs and GPCRs both possess seven transmembrane (7TM) domains, they exhibit opposing membrane topologies. This structural divergence has led to ongoing debate regarding the classification of ORs within Family A [33]. In this study, based on phylogenetic relationships and conserved domain analysis, PaltOR-1 and PaltOR-2 were provisionally assigned to the opsin subfamily. A total of 26 biogenic amine receptors were identified in *P. alternatus*. Phylogenetic analysis and structural similarity clustering classified these receptors into five categories: tyramine receptors (TARs), dopamine receptors (DopRs), octopamine receptors (OARs), muscarinic acetylcholine receptors (mAChRs), and 5-hydroxytryptamine receptors (5-HTRs). In this subfamily, PaltTAR-1~5 were analyzed as tyramine-like receptors. Three octopamine receptors (PaltOAR-1~3) were characterized in this subfamily. Notably, 5-HTR represented the most abundant subtype, with these receptors designated as Palt5-HTR-1 to Palt5-HTR-9. PaltmAChR-1 to PaltmAChR-5 were identified as muscarinic acetylcholine receptors and displayed high sequence homology to mAChR genes in other mite species. The neuropeptide and protein hormone receptors are the largest subfamily in Family A from *P. alternatus*. It was found that 34 neuropeptide and protein hormone receptors genes were subdivided into 14 subgroups, including sulfakinin receptor (SKR), ryamide receptor (Rya), relaxin receptor (RXFP), and CXC chemokine receptor 4 (CXCR4); two pyrokinin receptors (PKRs) and type-1 angiotensin II receptor (AT2R); and three adipokine chormone receptors (AKHRs), neuropeptide Y receptor (NPYR), and adenosine receptor (ADOR). Moreover, one ecdysis-triggering hormone receptor (ETHR) identified in mites through sequence alignment showed 100% similarity to the ETHR (AZL90164.1) of *P. citri.* Eight receptors (PaltTKR-1 to PaltTKR-8) were identified as orthologues of tachykinin receptor. PaltADR-1 and PaltADR-2 were closely related to *L. deliense* (RWS29702.1 and RWS24614.1) and *D. tinctorium* (RWS02772.1); they were identified by us as adrenergic receptors (ADRs). PaltAstAR-1 was characterized as an A Allatostatin-A receptor (AstAR), and it shares a sequence similarity of 100% with XP_015789363.1 (*T. urticae*).

**Figure 1:**
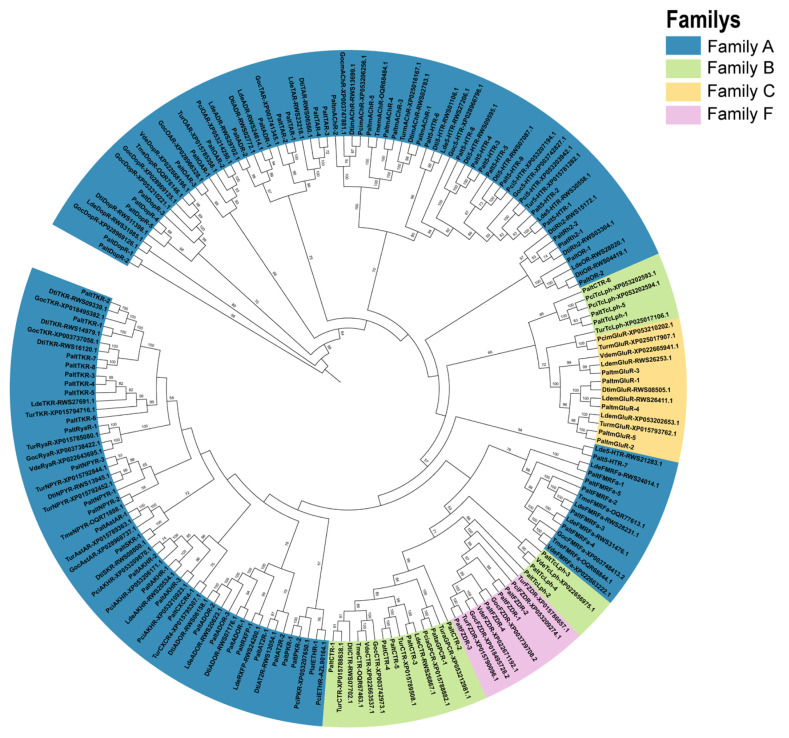
Phylogenetic maximum-likelihood tree of GPCR families from *P. alternatus* and other mite species.

The 12 identified Family B GPCRs were classified into three subgroups, with six assigned to Subfamily B1 as calcitonin receptors (CTRs). Adhesion-G-protein-coupled receptor (aGPCR) and latrophilin (TcLph) were classified under Subfamily B2, PaltaGPCR-1 belonged to the adhesion-G-protein-coupled receptor, and five TcLphs (PaltTcLph-1 to PaltTcLph-5) were identified in *P. alternatus*. Subfamily B3 exclusively comprises methuselah receptors. However, no members of this subfamily were identified in mites. All five Family C GPCRs were metabotropic glutamate receptors (mGluRs), showing high homology to orthologs in *L. deliense* (RWS26253.1, RWS26411.1), *V. destructor* (XP_022665941.1, XP_022665942.1), and *T. urticae* (XP_015793762.1). Moreover, Gamma-aminobutyric acid B (GABA-B) and bride of sevenless (boss-type) receptors were not detected. In the present work, four putative *P. alternatus* GPCRs were identified as frizzled receptors (PaltFZD-1 to PaltFZD-4), exhibiting significant homology to FZD receptors in *T. urticae*, *V. destructor*, and *G. occidentali* (Figure 1).

### 2.2. Identification of Conserved Motifs and Domain Analysis

**Figure 2:**
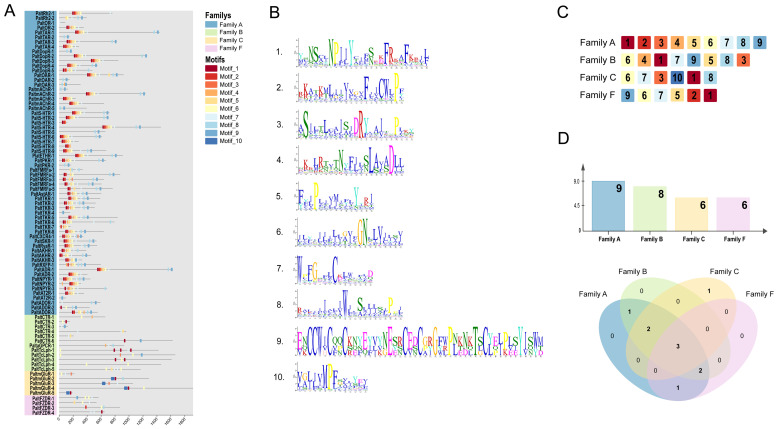
The motif compositions of the GPCR proteins and their distribution in *P. alternatus*. (**A**) The identified conserved motifs of the 85 GPCR proteins in *P. alternatus.* (**B**) The sequences of the 10 motifs detected by the MEME online tool. (**C**) The number of conserved motifs identified in each GPCR family. (**D**) A Venn diagram of the motifs detected in domain subfamilies A, B, C, and F (https://www.bioinformatics.com.cn/, accessed on 23 January 2025).

To further investigate the evolutionary diversification of GPCRs in *P. alternatus*, we analyzed the conservation of motifs across 85 GPCR proteins using the MEME suite. A total of 10 distinct motifs were identified (Figure 2A,B). Among the four GPCR families, Family A exhibited the highest number of conserved motifs. All four families shared three highly conserved motifs, Motif 1, Motif 6, and Motif 7, indicating that these motifs may be related to the 7tm_GPCRs domain. In addition to these shared motifs, Families A, B, and C contained two exclusive motifs (Motifs 3 and 8). Notably, some motifs were exclusively present in specific groups. Motif 2 was exclusively distributed in Families A and F. Motif 10 was uniquely identified in Family C (Figure 2C,D), suggesting potential divergence in evolutionary relationships within this family or specific functions.

**Figure 3:**
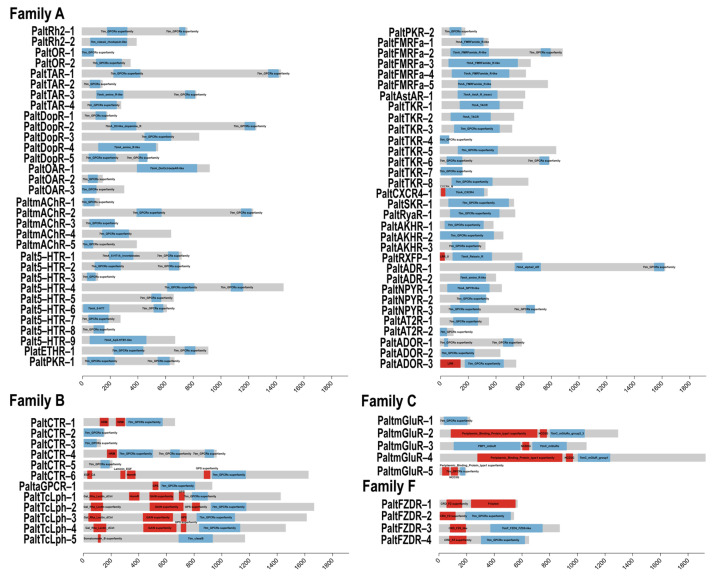
A schematic diagram of the *P. alternatus* GPCR domain. Blue boxes indicate the 7TM-GPCRs domain. Red boxes indicate the other domain.

Nearly all *P. alternatus* GPCR proteins identified in this study possessed at least one 7TM-GPCR superfamily domain (Figure 3). Sequence comparison suggests that GPCR proteins are highly conserved. Furthermore, over 80% of Family B, C, and F GPCRs exhibited multi-domain architectures, incorporating functional domains such as HRM, GPS, NCD3G, CRD_FZ, and GAIN. In contrast, such multi-domain configurations were rarely detected in Family A GPCRs.

### 2.3. Expression Profiles of GPCR Genes in Different Physiological States

To further investigate the potential biological functions of GPCRs in *P. alternatus*, we employed RT-qPCR to analyze the expression levels of 85 GPCR genes in *P. alternatus* across three distinct physiological states. The results show that all of these 85 GPCR genes were expressed. Only the *PaltmAChR-5* gene exhibited no statistically significant differences in expression levels across the three states. Since the physogastric stage harbors immature developmental stages, including larvae and nymphs, compared to this state, 43 genes were statistically significantly upregulated in the other two states, while 41 GPCR genes were significantly downregulated. *PaltOR-1* showed significantly higher expression in the phoretic compared to the viviparous mite, whereas *PaltOR-2* exhibited no statistically significant differences between these two states. Therefore, we hypothesized that *PaltOR-1* might have a potential regulatory role in response to the termination of phoresy. Meanwhile, *PaltOR-2* may play an important role in olfactory function throughout the adult lifespan of *P. alternatus*. These upregulated genes may participate in the olfactory recognition of environmental volatiles in *P. alternatus*, and we will prioritize their functional validation in subsequent investigations.

**Figure 4:**
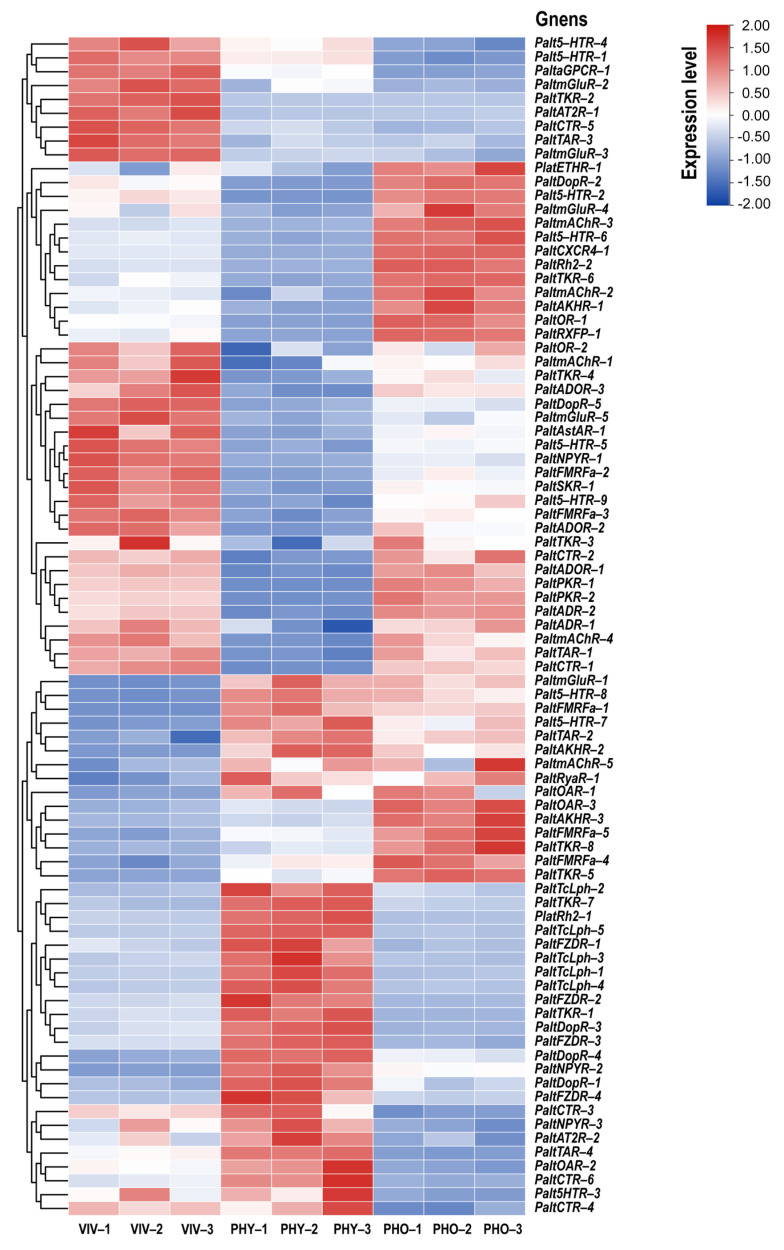
A heatmap showing the relative expression levels of 85 GPCR genes in *P. alternatus* across three distinct physiological states: 72 h developed physogastric state (PHY), viviparous state (VIV), and phoretic state (PHO). Rows were scaled by Z-score normalization (TBtools) to highlight stage-specific expression patterns. The color gradient reflects standardized expression levels (red: high; blue: low). Hierarchical clustering was applied to rows and columns using Euclidean distance and complete linkage.

**Figure 5:**
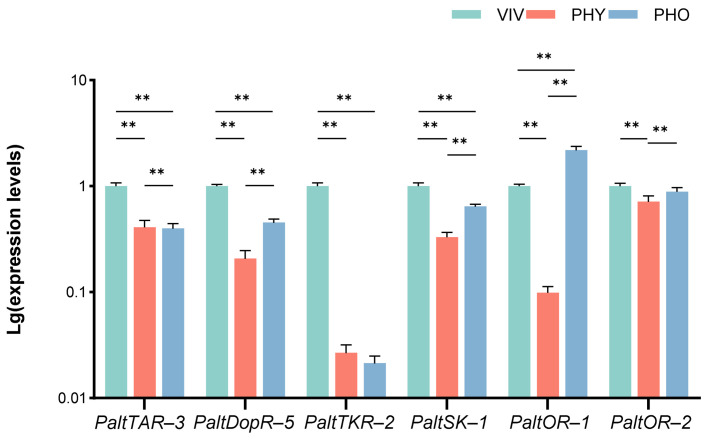
The relative expression levels of the upregulated genes in viviparous mites (VIV), 72 h developed physogastric mites (PHY), and phoretic mites (PHO). Data are presented as the means ± standard errors (SEM), and statistical comparisons were based on Student’s *t*-tests. *p* < 0.01 (**).

In viviparous mites, thirteen neuropeptide and protein hormone receptors were identified within the subset of upregulated genes. *PaltTKR-2* exhibited 37.11-fold and 46.96-fold upregulated expression compared to the physogastric and phoretic states, respectively. In physogastric, 24 candidate GPCR genes were significantly upregulated, and 34 genes were significantly downregulated. All Latrophilin and frizzled receptor genes were highly expressed in this state. *PaltCirl-4* exhibited 10.00-fold and 14.89-fold upregulation compared to the other two states, respectively. Compared to the phoretic state, *Palt5-HTR-3*, *PaltNPYR-3*, *PaltCTR-3*, and *PaltCTR-4* exhibited upregulated expression levels in both the viviparous and physogastric states, though no statistically significant differences were observed between these two physiological states. Moreover, 41 tested GPCR genes were significantly differentially expressed in the phoretic state. Among these, 22 genes exhibited statistically significantly upregulated expression levels, and 19 genes were significantly downregulated. In addition, six GPCR genes (*PaltDopR-4*, *Palt5-HTR-7*, *Palt5-HTR-8*, *PaltFMRFa-1*, *PaltTKR-7*, *PaltAKHR-2*, and *PaltNPYR-2*) were highly expressed in both the physiological and phoretic states, with significantly higher expression levels in the physiological compared to the phoretic mite (Figure 4 and Figure 5).

Based on previous phylogenetic, structural, and expression pattern analyses, PaltOR-1 and PaltOR-2 were identified as odorant receptors (Figure 1, Figure 2 and Figure 3). *PaltDopR-5*, *PaltSKR-1*, *PaltTAR-3*, and *PaltTKR-2* exhibited elevated expression during the viviparous mite stage, with functional validation of homologous protein receptors in olfactory signaling pathways across other insects [34,35,36,37]. Therefore, we selected these six receptors from the 85 GPCR candidates to investigate their binding capabilities to five major HIPVs from *P. massoniana* via molecular dynamics simulations (Figure 5).

### 2.4. Molecular Dynamics Simulation

**Figure 6:**
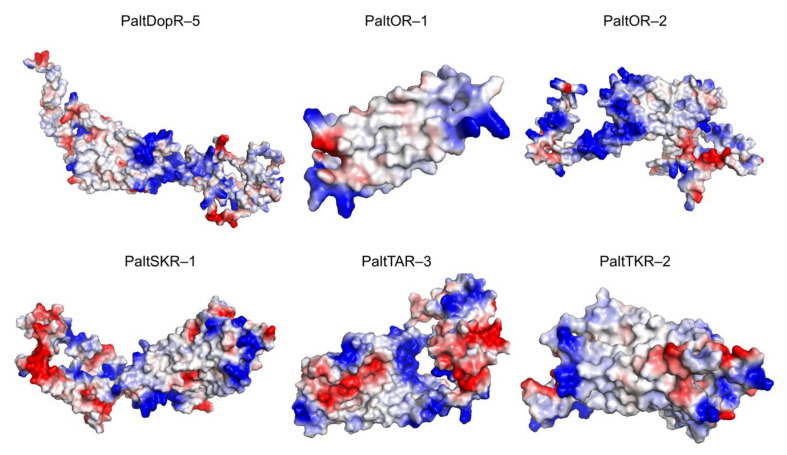
Three-dimensional structure model of docking protein in *P. alternatus.* Red indicates negative electrostatic potential regions (negatively charged), blue represents positive electrostatic potential regions (positively charged), and white corresponds to neutral regions.

**Figure A1:**
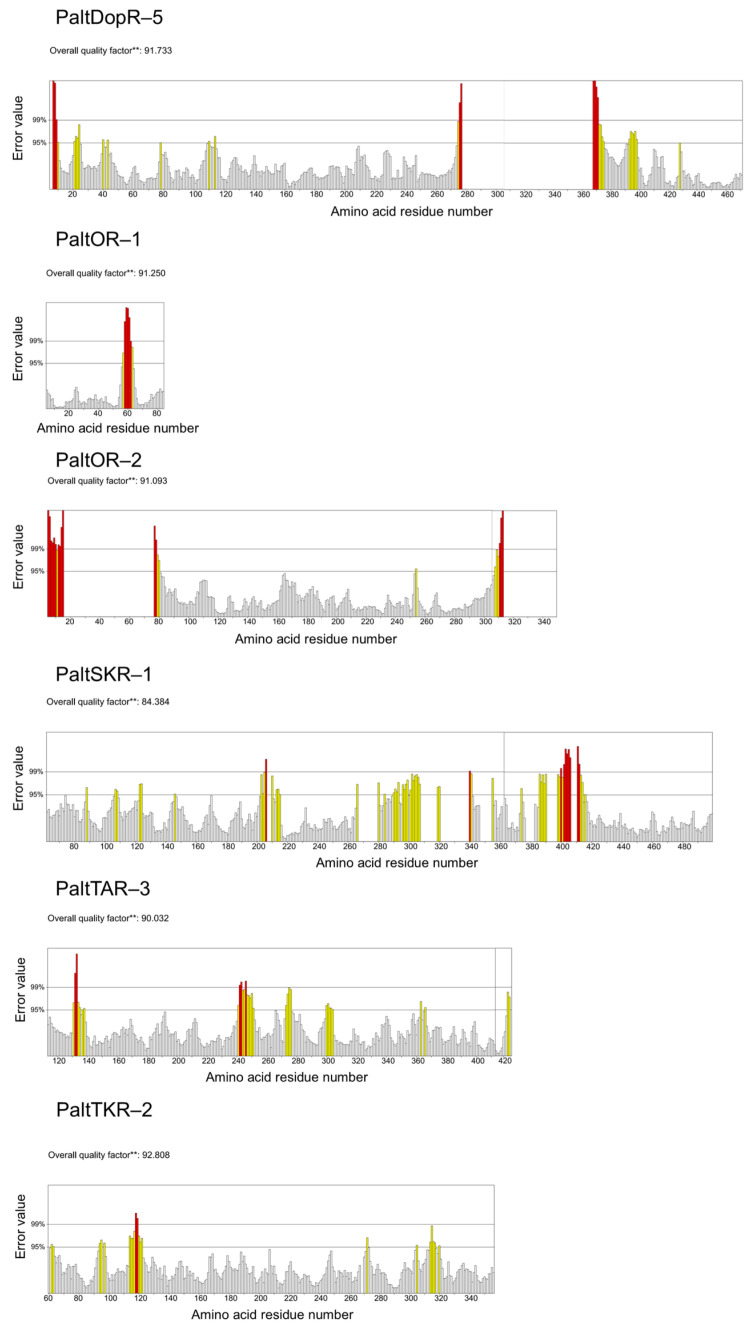
ERRAT assessment results of protein models in *P. alternatus*. ** *p* < 0.01 (reject null hypothesis).

**Figure A2:**
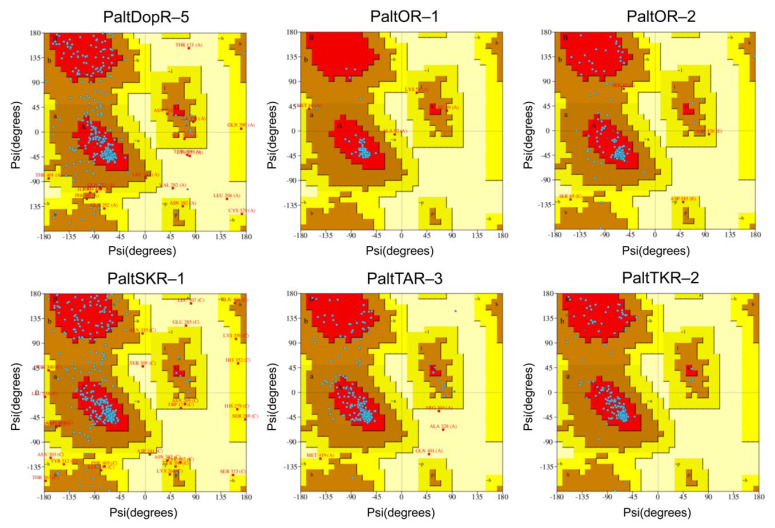
Ramachandran plots of protein models in *P. alternatus.* Note: the red area is the optimal area, the brown yellow area is the permissible area, the bright yellow area is the barely reasonable area, and the earthy yellow area is the unreasonable area.

Three-dimensional models of PaltDopR-5, PaltOR-1, PaltOR-2, PaltSKR-1, PaltTAR-3, and PaltTKR-2 were constructed using AlphaFold2. Based on the structural features of the protein, these charged regions may contribute to enhanced binding stability with ligands (Figure 6). The structural quality of these models was rigorously assessed based on the ERRAT score. PaltSKR-1 exhibited an ERRAT score of 84.384, while the remaining five protein models demonstrated high structural reliability, with scores all exceeding 90 (Figure A1). PROCHECK-generated Ramachandran plots confirmed high stereochemical quality across the protein models, with PaltOR-1 showing 92.5% of residues in the most favored regions, 2.5% in allowed regions, 5% in generously allowed regions, and no residues in disallowed regions. PaltOR-2 exhibited 91.8% of residues in the most favored and allowed regions combined, whereas PaltSKR-1 displayed 90.3% in these favorable regions. The remaining three proteins all surpassed 90% of residues in the most favored and allowed regions (Figure A2). Collectively, over 90% of residues across all models resided within allowed regions, confirming the reliability of the protein structures. These results suggest the structural plausibility of the models, supporting their suitability for downstream functional analyses.

Molecular docking analyses were conducted to investigate the interactions between the protein receptors and five HIPVs from *P. massoniana*. Binding energy values were used to quantify ligand–receptor binding capacity, with a threshold of −5.0 kcal/mol indicating stable interactions during molecular docking, where lower values denote stronger binding affinities. The results demonstrate that PaltSKR-1 exhibited the lowest binding energies with α-pinene, β-pinene, and longifolene, reaching −5.06 kcal/mol, −5.22 kcal/mol, and −5.73 kcal/mol, respectively. Furthermore, caryophyllene showed optimal binding to PaltTKR-2 at −5.80 kcal/mol, while phellandrene displayed the strongest interaction with PaltOR-1 at −5.83 kcal/mol. PaltTAR-3 exhibited weak binding affinities with all ligands, as evidenced by the binding energies failing to meet the stability threshold of −5.0 kcal/mol (Table 2).

**Figure 7:**
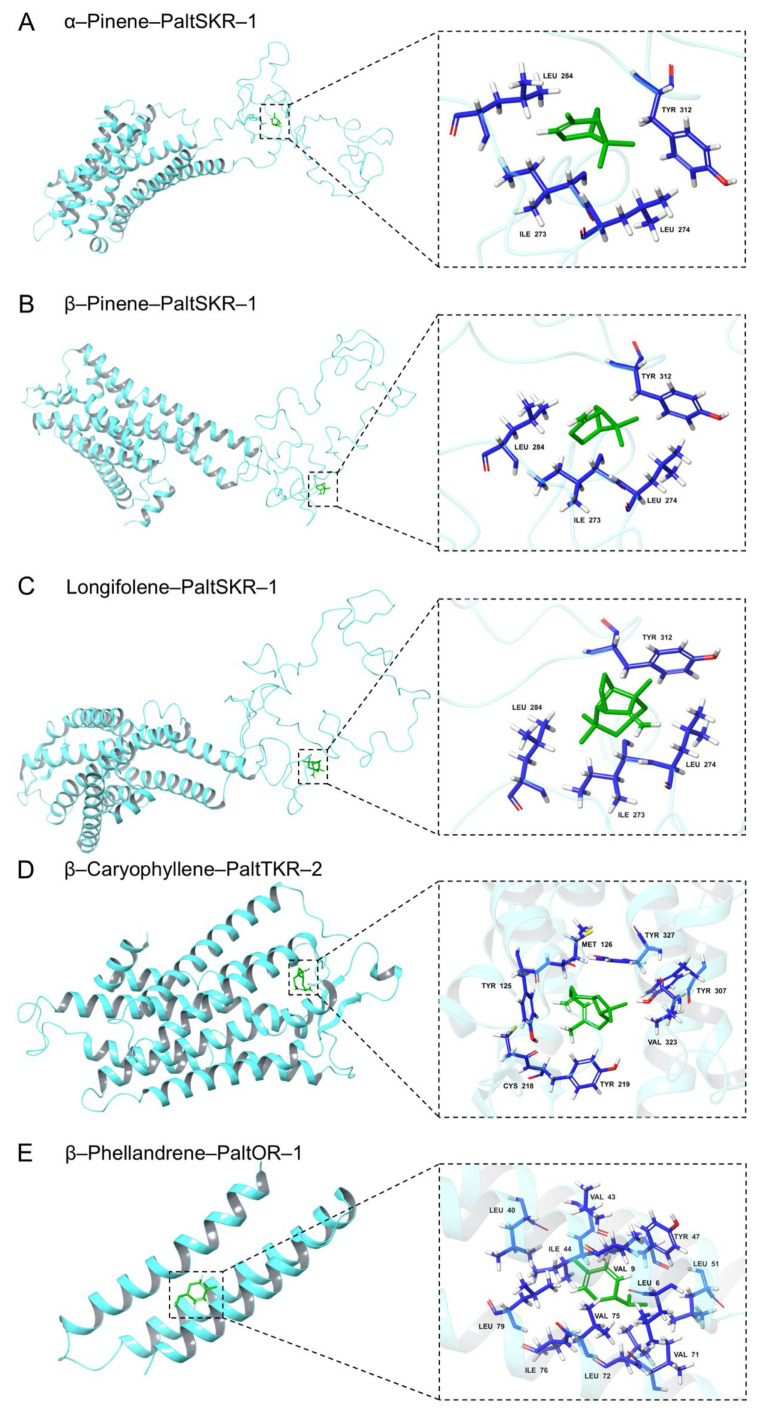
Molecular docking simulations of GPCR proteins with five active ligands. (**A**) Interaction diagram of α-Pinene–PaltSKR-1. (**B**) Interaction diagram of β-Pinene–PaltSKR-1. (**C**) Interaction diagram of longifolene–PaltSKR-1. (**D**) Interaction diagram of β-Caryophyllene–PaltTKR-2. (**E**) Interaction diagram of β-Phellandrene–PaltOR-1.

Based on the binding energy data, the protein models demonstrating the strongest binding affinities with each of the five ligands were selected for visualization analysis. Hydrophobic interactions were identified as the predominant binding forces between the protein models of *P. alternatus* GPCRs and ligands. For PaltSKR-1, critical residues mediating interactions with α-pinene, β-pinene, and longifolene included ILE 273, LEU 274, LEU 284, and TYR 312. In contrast, caryophyllene binding to PaltTKR-2 involved residues TYR 125, MET 126, CYS 218, TYR 219, TYR 307, VAL 323, and TYR 327. The hydrophobic network between PaltOR-1 and phellandrene comprised LEU 6, VAL 9, LEU 40, VAL 43, ILE 44, TYR 47, LEU 51, VAL 71, LEU 72, VAL 75, ILE 76, and LEU 79 (Figure 7). We found that PaltSKR-1 efficiently binds to diverse ligands while maintaining strict conservation of key catalytic residues, suggesting that its substrate adaptability may be mediated through an entropic compensation mechanism. Overall, these results suggest that specific GPCRs are functionally implicated in mediating olfactory recognition and chemosensory signaling pathways in *P. alternatus*. Nevertheless, further experimental validation is warranted.

## 3. Discussion

Numerous physiological processes in insects are tightly regulated by GPCRs. Therefore, by conducting bioinformatic analysis to discover genes in *P. alternatus* and elucidate their potential physiological functions, we aimed to provide a foundation for developing sustainable methods to biocontrol *M. alternatus*. In this study, a total of 85 GPCR genes were identified from the transcriptome of *P. alternatus*, which were categorized into the A, B, C, and F families, with 64, 12, 5, and 4 members, respectively. These protein receptors mostly possess a relatively similar structural diversity, and this gives insight into their evolutionary and potentially functional roles. Compared to GPCRs in other insect species, some duplication and missing events occurred in mite GPCR genes. In both *T. urticae* and *P. alternatus*, ETHR, RyaR, and AstAR are present with single copies [21]. However, SIFamide, pigment dispersing factor, and proctolin were not identified, whereas five FMRFa-related homologous receptors were detected in *P. alternatus*. FMRFa has been reported to modulate startle-induced locomotor activity and adaptive sleep following heat stress in *D. melanogaster*, while also regulating spontaneous locomotion in *Apolygus lucorum* [38,39,40]. Therefore, the expansion of FMRFa might contribute to the perception of environmental stimuli and the generation of adaptive behavioral responses among *P. alternatus.* In addition, there were gene repeats in mites for 5-HTR, mAChR, and TKR. These GPCRs can play a role in feeding behavior [41], intraspecific interactions [33], and other physiological processes [42,43]. *P. alternatus* retains only a single R-type opsin, suggesting reduced reliance on vision due to functional redundancy elimination or niche specialization [44]. This adaptation correlates with its dark bark-dwelling behavior and distinct positive phototaxis, which is consistent with its role in population dispersal [10]. Overall, we speculate that gene expansion and loss in *P. alternatus* underlie its adaptive evolution and enhanced environmental responsiveness.

To shed some light on the role that GPCRs play in *P. alternatus*, an expression profile analysis of these receptor genes was performed. In the physogastric state, 24 candidate GPCR genes exhibited significant upregulation, with all TcLph and FZD genes showing elevated expression in this state. Previous studies have demonstrated that RNA-interference (RNAi) knockdown of TcLph gene transcripts in female *Tribolium castaneum* induces ovarian atrophy and significantly impairs fecundity [45], while FZD receptors may be involved in insect growth through the transduction of critical developmental signaling pathways [40]. These receptors may be associated with reproductive processes and growth and development in *P. alternatus*. Phoresy is a temporary symbiosis where one life stage of a mite attaches to another insect for dispersal. In this state, mites cease feeding and suspend ontogenetic development [46]. During the transition from the active to phoretic state in *P. alternatus*, 41 genes exhibited significant expression changes, with 22 upregulated and 19 downregulated. Among these, *PaltTAR-4* and *PaltTKR-1* were downregulated 4.54-fold and 3.97-fold, respectively, while *PaltTKR-2* exhibited a dramatic 46.96-fold reduction in expression. The TKR plays a critical role in regulating trehalose homeostasis, reducing body weight, and enhancing resistance to starvation and oxidative stress in insects [41,47,48], indicating that these GPCRs might be involved in mite transition to the phoretic state and internal adaptive regulation. Furthermore, two ADRs, as well as the ETHR, CXCR4, and RXFP, exhibited significant upregulation. These receptors likely serve critical roles in modulating immune responses, energy metabolism, and stress adaptation mechanisms to enhance survival during phoresy. Similar results were reported in other insect species [49,50].

In insects, sulfakinin (SK) is predominantly expressed in a limited subset of neurons and neurosecretory cells localized within the brain [51]. It exerts diverse biological functions through activation of the SKR, including regulating feeding behavior and molting processes [36] and modulating mating and aggressive behaviors [52]. Notably, the SKR also appears to be involved in the regulation of odor preference [53]. In *P. alternatus*, the expression of this receptor gene was significantly upregulated during the viviparous state. In conclusion, we suggest that SKR may be involved in the olfactory perception mechanisms of mite, potentially mediating odorant detection and signal transduction critical for host-seeking behaviors.

HIPVs are specific volatile compounds released by host plants in response to insect damage. These compounds serve multiple ecological functions in mediating tritrophic interactions among plants, herbivores, and natural enemies, including priming plant defense mechanisms and acting as critical chemical cues for predators to locate prey and hosts [54]. *M. alternatus* is a serious wood borer of pine trees and is a widespread vector of pine wilt disease in Asia [55]. Adult feeding induces differential expression of terpene synthase-related genes in the metabolic pathways of *P. massoniana*, leading to elevated emissions of terpenoid compounds [55,56]. However, whether pine trees recruit natural enemies via HIPVs to establish indirect defense remains unresolved. Based on prior findings, we selected six candidate chemosensory receptors for molecular dynamics simulations. Our results reveal interactions between PaltSKR-1, PaltOR-1, and PaltTKR-2 in *P. alternatus* and major HIPVs from *P. massoniana*. Docking simulations suggest that specific GPCRs may bind to pine-derived HIPVs, potentially contributing to the chemosensory response of *P. alternatus*. The present results help us understand the mechanism underlying mite host location and highlight the need for additional work to explore the function of GPCRs in chemical ecological regulation.

## 4. Materials and Methods

### 4.1. Insect and Mite Rearing

The test *P. alternatus* was originally collected from Fujian Province, China (2018), and has since been reared in a laboratory. Mites were cultured on an artificial climate chamber at a temperature of 26 ± 0.5 °C and relative humidity of 90%, in complete darkness. *M. alternatus* eggs used to feed *P. alternatus* were obtained by cultivating adults of *M. alternatus* in Key Laboratory of Integrated Pest Management in Ecological Forests, Fujian Agriculture and Forestry University. Samples were collected from different developmental stages of *P. alternatus.* Three groups of 3 mg samples were separately collected: physogastric mites developed over 72 h, viviparous mites, and phoretic mites carried by *M. alternatus* for 15 days. Samples were flash-frozen in liquid nitrogen and stored at −80 °C until RNA extraction.

### 4.2. Identification and Analysis of P. alternatus GPCRs

The published *Dinothrombium tinctorium*, *Leptotrombidium deliense*, *Panonychus citri*, *Tetranychus urticae*, *Varroa destructor*, *Tropilaelaps mercedesae*, and *Galendromus occidentalis* GPCR genes were downloaded from NCBI. A local database was constructed from the transcriptome data of *P. alternatus* measured in the early stage of this experiment. BLASTP analysis was conducted using a minimum identity threshold of 60% and an *E*-value ≤ 1 × 10^−5^ to screen for putative GPCR genes of *P. alternatus* [29]. The putative GPCR genes were subjected to open reading frame (ORF) prediction using ORF Finder (https://www.ncbi.nlm.nih.gov/orffinder/, accessed on 14 February 2025), ultimately yielding the GPCR family protein sequences of *P. alternatus*.

Motif-based analysis of the protein sequences was performed using the MEME suite [30]. The conserved domain of candidate GPCR genes was analyzed using the conserved domain database (CDD) [31]. Based on the integrated analytical results above, family and subfamily classification of the GPCR genes was performed.

### 4.3. Phylogenetic Analysis

To determine the similarity and evolutionary relationships between the GPCRs of P. alternatus and 7 other mites, GPCR sequences were aligned with Clustal W (MAGE 7.0). Following sequence alignment, the phylogenetic tree was created using IQ-Tree v1.6.9 [32] with the maximum-likelihood (ML) method, using 1000 ultrafast bootstraps [57] and 1000 SH-aLRT replicates [58]; the best-fit partitioning schemes and substitution models were determined using ModeFinder [59]. The Interactive Tree Of Life was used to visualize and annotate the constructed phylogenetic tree [60]. Bootstrap values below 50% were not included in the visualization. 

### 4.4. RNA Extraction and cDNA Preparation

Total RNA was extracted from the samples using the HiPure Universal RNA Kit (Magen Biotech, Guangzhou, China) following the manufacturer’s instructions. The purity and integrity of total RNA were examined by using 2% agarose gel electrophoresis and the NanoDrop^TM^ One Microvolume UV-Vis Spectrophotometer. RNA was then reverse transcribed into cDNA using the HiScript^®^ Ⅲ 1st Strand cDNA Synthesis Kit (Vazyme, Nanjing, China). The quantity of cDNA produced was measured by using a spectrophotometer. Finally, the resulting cDNAs were diluted 10-fold with RNase-free ddH_2_O for use as templates in the subsequent RT-qPCR analyses and stored at −20 °C. The GPCR genes’ specific primers were designed based on each of the *P. alternatus* gene sequences using NCBI, and they are listed in Table A1.

### 4.5. Real-Time Quantitative PCR

RT-qPCR was performed on the QuantStudio 7 Pro Real-Time PCR System (Applied Biosystems, Foster City, CA, USA). The GAPDH (glyceraldehyde-3-phosphate dehydrogenase) gene was selected as an internal reference. Each individual RT-qPCR was conducted in a 20 μL reaction mixture containing 1.0 μL cDNA (100 ng/μL), 10 μL HRbio^TM^ qPCR SYBR Green Master Mix (Herui, Fuzhou, China), 7.0 μL RNase-free ddH_2_O, 1.0 μL forward primer (10 μM), and 1.0 μL reverse primer (10 μM). The RT-qPCR cycling parameters were as follows: 95 °C for 5 min, followed by 40 cycles of 95 °C for 10 s, 60 °C for 20 s, and 72 °C for 20 s. Then, the qPCR products were heated to 95 °C for 15 s, cooled to 60 °C for 1 min, and heated again to 95 °C for 1s to measure the melt curves. To ensure reproducibility, 3 biological replicates with 2 technical replicates were established. The 2^−ΔΔCt^ method was used to determine relative gene expression levels between different samples.

### 4.6. Structure Prediction and Molecular Docking

In this study, molecular docking validation was conducted to investigate the high-affinity binding of five *P. massoniana*-derived HIPVs to the GPCR receptors of *P. alternatus* (Table 3). AlphaFold2 was used for the prediction of the 3D structure [61]. Model quality assessment was conducted using SAVES v6.1 (https://saves.mbi.ucla.edu, accessed on 21 February 2025) and PROCHECK [62]. Binding pocket prediction was performed with the POCASA 1.1 [63]. Ligand molecules for docking were downloaded from the PubChem database (https://pubchem.ncbi.nlm.nih.gov/, accessed on 1 March 2025). Docking was performed using semi-flexible molecular docking in AutoDock Vina 4.2. A total of 100 poses per ligand were generated. The conformation with the lowest binding energy was ultimately selected as the representative pose [64]. Following docking, the optimal complex conformation was selected based on binding poses and energies for further analysis. Structural visualization analysis was carried out via PyMOL (Version 3.0.3). Two-dimensional structural visualization was achieved using Discovery Studio Visualizer^TM^ (v24.1.0.23298).

### 4.7. Data Analysis

Statistical analysis was conducted using SPSS 27.0 with an independent samples *t*-test and measuring variance (ANOVA) with Tukey’s post hoc test; differences were considered statistically significant when the *p* value was < 0.05.

## 5. Conclusions

In this study, we systematically identified 85 GPCRs from *P. alternatus* through comprehensive phylogenetic tree construction, protein structural analysis, and RT-qPCR expression profiling. In addition, molecular docking techniques further revealed that these GPCRs may mediate the mite’s perception of HIPVs from damaged host pine. The findings provide important clues for elucidating the biological functions of GPCRs in *P. alternatus* while establishing a theoretical foundation for developing novel biocontrol strategies targeting the egg stage of *M. alternatus*, thus contributing to the prevention of pine wilt disease.

## Figures and Tables

**Table 1:** Identification of 85 GPCRs in *P. alternatus*.

Receptor No. in the Text	Accession Number	Length (aa)	Receptor No. in the Text	Accession Number	Length (aa)
Family A			PaltTKR-6	XP_015794716.1	806
PaltRh2-1	RWS15172.1	775	PaltTKR-7	XP_003737058.1	166
PaltRh2-2	RWS03304.1	402	PaltTKR-8	RWS16120.1	649
PaltOR-1	RWS28020.1	88	PaltSKR-1	RWS08800.1	543
PaltOR-2	RWS04419.1	359	PaltCXCR4-1	XP_015795307.1	351
PaltTAR-1	RWS23218.1	1462	PaltRyaR-1	XP_015785080.1	553
PaltTAR-2	XP_003741345.1	152	PaltAKHR-1	RWS20534.1	393
PaltTAR-3	RWS23218.1	840	PaltAKHR-2	XP_053206171.1	465
PaltTAR-4	RWS06560.1	288	PaltAKHR-3	XP_053210923.1	334
PaltDopR-1	XP_028969126.1	187	PaltRXFP-1	RWS24200.1	605
PaltDopR-2	RWS31985.1	1282	PaltADR-1	RWS24614.1	1655
PaltDopR-3	XP_028969125.1	862	PaltADR-2	RWS29702.1	412
PaltDopR-4	OQR76146.1	560	PaltNPYR-1	XP_015792452.1	455
PaltDopR-5	RWS11398.1	482	PaltNPYR-2	OQR71898.1	342
PaltOAR-1	XP_015785358.1	939	PaltNPYR-3	XP_015792844.1	696
PaltOAR-2	XP_053214250.1	153	PaltAT2R-1	RWS08012.1	359
PaltOAR-3	XP_028966328.1	311	PaltAT2R-2	RWS13054.1	100
PaltmAChR-1	RWS02783.1	129	PaltADOR-1	RWS07176.1	601
PaltmAChR-2	XP_003747881.1	1256	PaltADOR-2	RWS06158.1	444
PaltmAChR-3	XP_053206256.1	240	PaltADOR-3	RWS20623.1	560
PaltmAChR-4	XP_025016167.1	654	Family B		
PaltmAChR-5	OQR68484.1	401	PaltCTR-1	XP_015788638.1	660
Palt5-HTR-1	RWS30558.1	733	PaltCTR-2	XP_053207328.1	151
Palt5-HTR-2	XP_015781282.1	723	PaltCTR-3	RWS26867.1	124
Palt5-HTR-3	XP_022654876.1	118	PaltCTR-4	XP_015789508.1	961
Palt5-HTR-4	XP_003742827.1	1481	PaltCTR-5	XP_022663537.1	208
Palt5-HTR-5	RWS07087.1	674	PaltCTR-6	XP_022645609.1	1625
Palt5-HTR-6	RWS01106.1	624	PaltaGPCR-1	XP_015788882.1	944
Palt5-HTR-7	XP_053203942.1	283	PaltTcLph-1	XP_053202594.1	1425
Palt5-HTR-8	RWS27206.1	166	PaltTcLph-2	XP_028966344.1	1665
Palt5-HTR-9	XP_053207194.1	685	PaltTcLph-3	XP_022656988.1	1612
PaltETHR-1	AZL90164.1	928	PaltTcLph-4	XP_022656975.1	1460
PaltPKR-1	XP_053210255.1	680	PaltTcLph-5	XP_053202593.1	1166
PaltPKR-2	XP_053207649.1	173	Family C		
PaltFMRFa-1	RWS24014.1	343	PaltmGluR-1	RWS08505.1	221
PaltFMRFa-2	XP_022663222.1	888	PaltmGluR-2	XP_053202653.1	1309
PaltFMRFa-3	RWS28231.1	653	PaltmGluR-3	RWS26253.1	1075
PaltFMRFa-4	RWS31476.1	618	PaltmGluR-4	RWS26411.1	1949
PaltFMRFa-5	OQR77613.1	779	PaltmGluR-5	XP_015793762.1	193
PaltAstAR-1	XP_015789363.1	614	Family D		
PaltTKR-1	RWS14979.1	597	PaltFZDR-1	XP_015786657.1	572
PaltTKR-2	RWS09339.1	532	PaltFZDR-2	XP_053209274.1	544
PaltTKR-3	XP_015790474.1	518	PaltFZDR-3	XP_015790096.1	886
PaltTKR-4	RWS27691.1	159	PaltFZDR-4	XP_003739708.2	660
PaltTKR-5	XP_053211289.1	853			

**Table 2:** GPCR protein–ligand interaction energy calculated using molecular docking.

	α-Pinene	β-Pinene	Longifolene	β-Caryophyllene	β-Phellandrene
PaltDopR-5	−4.45	−4.56	−5.17	−5.37	−4.38
PaltOR-1	−4.37	−4.43	−5.62	−5.19	−5.83
PaltOR-2	−4.38	−4.38	−5.15	−5.17	−4.68
PaltSKR-1	−5.06	−5.22	−5.73	−5.35	−4.38
PaltTAR-3	−4.14	−4.05	−4.69	−4.99	−4.11
PaltTKR-2	−4.70	−5.00	−5.35	−5.80	−4.72

**Table 3:** Compounds for molecular docking.

Compounds	Molecular Formula	Structural Formula	CAS NO.
α-Pinene	C_10_H_16_	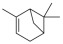	80-56-8
β-pinene	C_10_H_16_	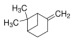	127-91-3
Longifolene	C_15_H_24_	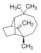	475-20-7
β-Caryophyllene	C_15_H_24_	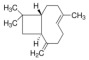	87-44-5
β-Phellandrene	C_10_H_16_	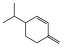	99-83-2

## Data Availability

The original contributions presented in this study are included in this article. Further inquiries can be directed to the corresponding author.

## Appendix A

**Table A1:** RT-qPCR primers for GPCR genes of *P. alternatus*.

Gene	Primer Name	Primer Sequences
*GAPDH*	GAPDH-F	ATCGAAAACGCAGCTTGCAG
	GAPDH-R	GTCGTGGTGCAGGACAAAAC
*PaltRh2-1*	PaltRh2-1-F	GTTAGGTATCACCGTATTGCCCT
	PaltRh2-1-R	GAAGCAGTAGATAGCCAAACGTC
*PaltRh2-2*	PaltRh2-2-F	TATGGTCAACGGATGTGCGTAA
	PaltRh2-2-R	GTCCTTAGTTGTGGTTGCATTGG
*PaltOR-1*	PaltOR-1-F	CATGACACTTTTCACCTATGCCC
	PaltOR-1-R	TAAATGATGGGGTTGAGGAGTGG
*PaltOR-2*	PaltOR-2-F	TGCTCTTTATAGTCGAGTTTGTGT
	PaltOR-2-R	GCAACAGCTTGACCATTTATTCG
*PaltTAR-1*	PaltTAR-1-F	GCCTTTATCTGTGGCGTACAATG
	PaltTAR-1-R	GGCTTGATACCTATCTACCGCAA
*PaltTAR-2*	PaltTAR-2-F	GTTCACATATCCTAGCCAGGTGA
	PaltTAR-2-R	CTCAGGTACTTGGCATTGTGATG
*PaltTAR-3*	PaltTAR-3-F	ATCCAGTTTACGTGGTGTTTGC
	PaltTAR-3-R	CTAGATCGAGCAAACAGATGGC
*PaltTAR-4*	PaltTAR-4-F	TGTGTGAAAATGATTTGTGCCCT
	PaltTAR-4-R	ATTCACCAAACCCATCACCATTG
*PaltDopR-1*	PaltDopR-1-F	CTTCTCATGCCGTCTGTTTAAGC
	PaltDopR-1-R	AAAACCAGGCAGGCTTAAGACT
*PaltDopR-2*	PaltDopR-2-F	CTGAACAGCAGCCTTTTAACACC
	PaltDopR-2-R	GTCAGTTACTTCTTGCGAATCCG
*PaltDopR-3*	PaltDopR-3-F	GCAGTAAGCCCAGAAAATAAGCC
	PaltDopR-3-R	CACCATGACTACCAAAGGACCAT
*PaltDopR-4*	PaltDopR-4-F	TGATGCCATTTGGTGTTATTGCT
	PaltDopR-4-R	TCAGAATTGATGCAGTACAGGCA
*PaltDopR-5*	PaltDopR-5-F	TCAATAAAGCCGCAAGTGAATGG
	PaltDopR-5-R	TTTCAGCTTCAACTTGCATTTGC
*PaltOAR-1*	PaltOAR-1-F	CCGTGTTGTCTGCATATTCGTC
	PaltOAR-1-R	AGTCGGGAAACAATGGTGATGA
*PaltOAR-2*	PaltOAR-2-F	CGAAGAAGATGGTGTCAACGC
	PaltOAR-2-R	ACTTTTCCACCACTAGACGGAG
*PaltOAR-3*	PaltOAR-3-F	CATGCCGTAGCTATGAGTTCAAC
	PaltOAR-3-R	CTAGGTCCAGTTCGAAAACAAGG
*PaltmAChR-1*	PaltmAChR-1-F	GACATGGACACCGTATAATGTGG
	PaltmAChR-1-R	GCATAGCAAAGGGGATTTACAGTG
*PaltmAChR-2*	PaltmAChR-2-F	GAAGCATCTTTTCCAGAGATGGC
	PaltmAChR-2-R	GAGCTTGAGTACACAGGTGATCT
*PaltmAChR-3*	PaltmAChR-3-F	GGCTGAACAGGAAGTAGTTGTTG
	PaltmAChR-3-R	GAGACAGTGGAGATGGTGTTCAT
*PaltmAChR-4*	PaltmAChR-4-F	GTTCGACGTACTACTAGACGAGC
	PaltmAChR-4-R	AGTACGTTCACCCTCAATGTAGG
*PaltmAChR-5*	PaltmAChR-5-F	CGAGTAATGGCCACACATATCAC
	PaltmAChR-5-R	CTGTGAGGTAGTAGGTGATTGCA
*Palt5-HTR-1*	Palt5-HTR-1-F	ATTGCCGCTGTTTTAAGTGACC
	Palt5-HTR-1-R	AAAACGCCTAATGTCCATTGGC
*Palt5-HTR-2*	Palt5-HTR-2-F	GCTGGCAACTTGGTTCAGAATT
	Palt5-HTR-2-R	TCCAGTACTTCGACCCTGACTAT
*Palt5-HTR-3*	Palt5-HTR-3-F	TGTAATGGGTGTTTTCGGCA
	Palt5-HTR-3-R	ACAACATGAGGAATGGCATCAC
*Palt5-HTR-4*	Palt5-HTR-4-F	AAGAGCAGTGAGGTTCCTACAAG
	Palt5-HTR-4-R	GTGGACAAAGACCAATCTGCTAC
*Palt5-HTR-5*	Palt5-HTR-5-F	CTTAACTTGGTTGGGGGTAATCG
	Palt5-HTR-5-R	GGACGCAATGGAACTGCTTAAT
*Palt5-HTR-6*	Palt5-HTR-6-F	GCATTAAGTGATCTTGGTGTGGC
	Palt5-HTR-6-R	CAGCAAACGACATCTAAACCCAG
*Palt5-HTR-7*	Palt5-HTR-7-F	CACCGTGTTTAAATCATCTCGTGT
	Palt5-HTR-7-R	ACAATTCCATTTCCAAAAACTGCG
*Palt5-HTR-8*	Palt5-HTR-8-F	CCGCAAACTAGTAGTGTAGACCA
	Palt5-HTR-8-R	ACTTTGAAAATCGCTTTTTGCCG
*Palt5-HTR-9*	Palt5-HTR-9-F	GCAGTGGTCAGTTCTTGCATTAG
	Palt5-HTR-9-R	GGCTTGAGGGCTAATATTAATGGC
*PlatETHR-1*	PlatETHR-1-F	TTTCACACTGGCTTTGCTATGC
	PlatETHR-1-R	CATACACATACAGCTGAACGCG
*PaltPKR-1*	PaltPKR-1-F	CCAATATCGGGATCCCAGTCTTT
	PaltPKR-1-R	TCCATGATTTGAATCGCCAGGA
*PaltPKR-2*	PaltPKR-2-F	TTTGAGCTTCAGTAGGCTGAGTG
	PaltPKR-2-R	AAAGATGTTAGCTGCCGTGGTA
*PaltFMRFa-1*	PaltFMRFa-1-F	TGATGCCATTTGGTGTTATTGCT
	PaltFMRFa-1-R	TCAGAATTGATGCAGTACAGGCA
*PaltFMRFa-2*	PaltFMRFa-2-F	ACGGTATAGTAAGACATGGGCTG
	PaltFMRFa-2-R	CCATGTAACGCCATACACCTAGT
*PaltFMRFa-3*	PaltFMRFa-3-F	TGGCACAGAAAGTATTGCTTCG
	PaltFMRFa-3-R	AACAAGACAAGAATGCCGGAAG
*PaltFMRFa-4*	PaltFMRFa-4-F	GCCTCTTGTCAGACAACTGTTTC
	PaltFMRFa-4-R	AAAGCATGCGGTTGATGTACAG
*PaltFMRFa-5*	PaltFMRFa-5-F	CCTTGTTACTTGCAGGTCAAAGG
	PaltFMRFa-5-R	GTACTCTGATGGAGAAATGCAGC
*PaltAstAR-1*	PaltAstAR-1-F	GGTCATAGTAGTGGTTTGCAATCC
	PaltAstAR-1-R	CTGAAGCAGTAAACGGTACACA
*PaltTKR-1*	PaltTKR-1-F	CCAGATGGTGTAGCACAAGAATC
	PaltTKR-1-R	CCCCATAGTACAATGGCTACTCG
*PaltTKR-2*	PaltTKR-2-F	TGGTGACTCTCGTGTAATCTGT
	PaltTKR-2-R	AGGCACCATGTACGTTACAAGT
*PaltTKR-3*	PaltTKR-3-F	AATGTTAGAACGGCATACCAGC
	PaltTKR-3-R	AATGTGCACAGCTTGGAATGTC
*PaltTKR-4*	PaltTKR-4-F	GTACTTCAATGTGCACAGCTTGG
	PaltTKR-4-R	CAATGTTAGAACGGCATACCAGC
*PaltTKR-5*	PaltTKR-5-F	GCCTCTGCGGTTACTACTGTTAA
	PaltTKR-5-R	TGTAGAGTCGAAACTAGCATCCG
*PaltTKR-6*	PaltTKR-6-F	CTGGGTGCTTCCATACTTTCTCT
	PaltTKR-6-R	GACCACTAAATAACGATCTGCTGC
*PaltTKR-7*	PaltTKR-7-F	TGCAACGTAACGGTCGAAAC
	PaltTKR-7-R	TGGAAAGCTGGCCTTATGGG
*PaltTKR-8*	PaltTKR-8-F	CGACCGTTACGTTGCAATTGTA
	PaltTKR-8-R	GGCATAGCTACAATCAACGAACC
*PaltCXCR4-1*	PaltCXCR4-1-F	GCCCACCATCTACTTCATCATCT
	PaltCXCR4-1-R	TGTGATGACAAAGAGGAGGTCAG
*PaltRyaR-1*	PaltRyaR-1-F	CAGTATTGGCCTTTTGGTTCTGTC
	PaltRyaR-1-R	TCTGTCTATGCTGATTGCCACA
*PaltAKHR-1*	PaltAKHR-1-F	TCGAGCAGCACCTCAAAATACA
	PaltAKHR-1-R	CTGTTCGGTTAAAGGCAAATCCTC
*PaltAKHR-2*	PaltAKHR-2-F	GCATCTCGCAATCAGTGACTTAC
	PaltAKHR-2-R	TAGAGCCATATGTCACAAGCACC
*PaltAKHR-3*	PaltAKHR-3-F	TCAAGAAGGTCAGCTAAATGGCA
	PaltAKHR-3-R	ACAACACCAGCAAATTACCAGTT
*PaltRXFP-1*	PaltRXFP-1-F	GACAAGTAGGTCGTCAAATGGCT
	PaltRXFP-1-R	GGGAAGAATAAATGCTGCAGTCC
*PaltADR-1*	PaltADR-1-F	GATGGAATCAGGGTTCGGCTTAA
	PaltADR-1-R	CGTCGTTTTGCCCTCTTGATTAG
*PaltADR-2*	PaltADR-2-F	CTTACGAGTTTGGCCATTTGGAG
	PaltADR-2-R	GACTTGTGATTGCCCAGTACCTA
*PaltNPYR-1*	PaltNPYR-1-F	TAGTAGCTGGTCTAGAAGCCACA
	PaltNPYR-1-R	TGATCTCAATGGTTGGCACGAT
*PaltNPYR-2*	PaltNPYR-2-F	GGTGTTACGGGTAATGGTCTAGT
	PaltNPYR-2-R	AGAGGTGTTATTGGTACGGCAA
*PaltNPYR-3*	PaltNPYR-3-F	AAAACATCTGTGCCTTCTTCACA
	PaltNPYR-3-R	AGGTGGCCAATTGCAGAATATTG
*PaltAT2R-1*	PaltAT2R-1-F	AGCCACGGGAATTTAGAGTTGT
	PaltAT2R-1-R	CAATCCAAGGCACAAGCATCTC
*PaltAT2R-2*	PaltAT2R-2-F	GTCTACCAAGTTTCGAAGCCAAC
	PaltAT2R-2-R	CAAGTTGAAGCGTAGTTCGTCTG
*PaltADOR-1*	PaltADOR-1-F	GAGGCAGTGGGTACTTCTTTTTG
	PaltADOR-1-R	TGATAACTCGTATTATCCGCTGGG
*PaltADOR-2*	PaltADOR-2-F	GATAGTGGTTTAGGAGTGCACCA
	PaltADOR-2-R	TAACCGCCTAGATTTGAACCTCC
*PaltADOR-3*	PaltADOR-3-F	CTATGGCTGCCCCTAAAGAAATG
	PaltADOR-3-R	TGAAGTTCTGCCTGAGGGATATG
*PaltCTR-1*	PaltCTR-1-F	GTGGATTCCATCGAAGTGCAAC
	PaltCTR-1-R	GCTCCAACCATCAAAAGTAGCC
*PaltCTR-2*	PaltCTR-2-F	GGCAATTCTCATTATTGGCCAACTAT
	PaltCTR-2-R	ACCCCATCCAAGAATTACGTATTTG
*PaltCTR-3*	PaltCTR-3-F	TGCTGCAGTTAAAGCATCAATTACA
	PaltCTR-3-R	TTCCCAAATAAGATAAAACTTCTTGCA
*PaltCTR-4*	PaltCTR-4-F	GTTTGCTGGCTCGCTGAATTAT
	PaltCTR-4-R	ACTTGCTGTACCTCGTTAGTGG
*PaltCTR-5*	PaltCTR-5-F	TATCACTACGCGCTGCATCC
	PaltCTR-5-R	AATTCAGCGAGCCAGCAAAC
*PaltCTR-6*	PaltCTR-6-F	GACAGTGCCATTGTAAACCAGG
	PaltCTR-6-R	TATCATAGACGACTTCGCAGCC
*PaltaGPCR-1*	PaltaGPCR-1-F	TGTGCGAATTACATAAACCTCGA
	PaltaGPCR-1-R	ACTGTAGGAGCGATAGTAGCTT
*PaltTcLph-1*	PaltTcLph-1-F	CTGGAATAGCGCAATGGTTCTG
	PaltTcLph-1-R	ACATTGAGCGAATATTGGCTGC
*PaltTcLph-2*	PaltTcLph-2-F	AGGCAGTTTTCCTGTTAACCCA
	PaltTcLph-2-R	CAGGTGGGTTATGGTGCCTTAT
*PaltTcLph-3*	PaltTcLph-3-F	GAACACCATAAGTTTGCACCCC
	PaltTcLph-3-R	CAGCTACTTCCTGTGGTTGCTA
*PaltTcLph-4*	PaltTcLph-4-F	TTGAACAGCCATGTCCAGAAGA
	PaltTcLph-4-R	GGTTTTGAGCTTGGCATCCATT
*PaltTcLph-5*	PaltTcLph-5-F	GTTCGAAACACTTGTGCAGACA
	PaltTcLph-5-R	GGCACAATGCGGATTCCAATAA
*PaltmGluR-1*	PaltmGluR-1-F	CATGGAGATTTACAGGTGCCTTTT
	PaltmGluR-1-R	CCAAGTAATGTAGTGCCAACATTGT
*PaltmGluR-2*	PaltmGluR-2-F	TGGTTGGCTTTTGTACCCATCT
	PaltmGluR-2-R	AAGCCACATATGCAGACAGTGA
*PaltmGluR-3*	PaltmGluR-3-F	GATTTGCCTGGGGATTTATCGC
	PaltmGluR-3-R	GAGCCTGTATACCACCTTGAGG
*PaltmGluR-4*	PaltmGluR-4-F	TTTTTGCGAAGGAATGACGGTC
	PaltmGluR-4-R	TCTGTCTGACCAACCATCACTG
*PaltmGluR-5*	PaltmGluR-5-F	CTTTTCTGTTGCCCGCTCTAAC
	PaltmGluR-5-R	TTGCTTACGCCTGTTCTAAGGT
*PaltFZDR-1*	PaltFZDR-1-F	CCATGCAACTCAATCACAAGCA
	PaltFZDR-1-R	TGCATGGAGGTATGGGACTATC
*PaltFZDR-2*	PaltFZDR-2-F	AACAAGGAGCACAAGGAAATGC
	PaltFZDR-2-R	AGTCCTGCTTCTAGATACCAGGT
*PaltFZDR-3*	PaltFZDR-3-F	CCGGATTTTGGATTTGGTCTGG
	PaltFZDR-3-R	TGCGATGCTTTTTCCCATTCTC
*PaltFZDR-4*	PaltFZDR-4-F	GACCTTTGTGTGATACAGTGCG
	PaltFZDR-4-R	TGGCCCTTCCATACACATTTGA
